# Framing pictures: A conceptual framework to identify and correct for biases in detection probability of camera traps enabling multi‐species comparison

**DOI:** 10.1002/ece3.4878

**Published:** 2019-01-23

**Authors:** Tim R. Hofmeester, Joris P. G. M. Cromsigt, John Odden, Henrik Andrén, Jonas Kindberg, John D. C. Linnell

**Affiliations:** ^1^ Department of Wildlife, Fish, and Environmental Studies Swedish University of Agricultural Sciences Umeå Sweden; ^2^ Centre for African Conservation Ecology, Department of Zoology Nelson Mandela University Port Elizabeth South Africa; ^3^ Norwegian Institute for Nature Research Oslo Norway; ^4^ Grimsö Wildlife Research Station, Department of Ecology Swedish University of Agricultural Sciences Riddarhyttan Sweden; ^5^ Norwegian Institute for Nature Research Trondheim Norway

**Keywords:** animal characteristics, detectability, environmental variables, mammal monitoring, reuse of data, trail camera

## Abstract

Obtaining reliable species observations is of great importance in animal ecology and wildlife conservation. An increasing number of studies use camera traps (CTs) to study wildlife communities, and an increasing effort is made to make better use and reuse of the large amounts of data that are produced. It is in these circumstances that it becomes paramount to correct for the species‐ and study‐specific variation in imperfect detection within CTs. We reviewed the literature and used our own experience to compile a list of factors that affect CT detection of animals. We did this within a conceptual framework of six distinct scales separating out the influences of (a) animal characteristics, (b) CT specifications, (c) CT set‐up protocols, and (d) environmental variables. We identified 40 factors that can potentially influence the detection of animals by CTs at these six scales. Many of these factors were related to only a few overarching parameters. Most of the animal characteristics scale with body mass and diet type, and most environmental characteristics differ with season or latitude such that remote sensing products like NDVI could be used as a proxy index to capture this variation. Factors that influence detection at the microsite and camera scales are probably the most important in determining CT detection of animals. The type of study and specific research question will determine which factors should be corrected. Corrections can be done by directly adjusting the CT metric of interest or by using covariates in a statistical framework. Our conceptual framework can be used to design better CT studies and help when analyzing CT data. Furthermore, it provides an overview of which factors should be reported in CT studies to make them repeatable, comparable, and their data reusable. This should greatly improve the possibilities for global scale analyses of (reused) CT data.

## 1 INTRODUCTION

Obtaining reliable species observations is the key process underlying the study of animal ecology to facilitate wildlife conservation. Where researchers previously used to rely on direct observations and signs of animals, technological advances have expanded the toolbox. Recent years have seen an enormous increase in the number of studies that use camera traps (CTs) to detect animals (Burton et al., 2015; Rowcliffe & Carbone, 2008). CTs are mainly used to study terrestrial mammals, especially elusive species that are otherwise difficult to study (Burton et al., 2015). While their use is often primarily motivated by a desire to study a key species in a specific study site, increasingly CTs are seen as a potential tool for simultaneously investigating multiple species. The underlying assumption being that they are relatively unselective in which species they record, due to the passive infrared (PIR) sensors that trigger most modern‐day CTs (Rovero, Zimmermann, Berzi, & Meek, 2013). In addition, the dramatic increase in CT studies across the globe opens for (re)use of data for comparative studies across multiple seasons and sites (Scotson, Fredriksson, Ngoprasert, Wong, & Fieberg, 2017a; Steenweg et al., 2017). It is when moving from single species to communities or from single sites to diverse environmental conditions that caution is required when interpreting the data due to implications of the differences in detection among species, environments, and seasons.

There are already several reviews on how to apply CTs, what to consider in CT study design, and which types of CTs to use (e.g., O'Connell et al., 2011; Rovero & Zimmermann, 2016), and it has been acknowledged for some time that analyses should take the imperfect detection of CTs into account (Rowcliffe & Carbone, 2008; Tobler, Carrillo‐Percastegui, Leite Pitman, Mares, & Powell, 2008). A review by Burton et al. (2015) showed, however, that only a minority of studies actually follow through on this advice. Camera traps are often used to estimate relative abundance based on detection rates (Burton et al., 2015). However, many other factors apart from abundance also influence CT detection of animals including the size of the animal, its movement rate, the denseness of the vegetation, the presence of a trail in front of the CT, and the use of attractants (Cusack, Dickman, et al., 2015a; Hofmeester, Rowcliffe, & Jansen, 2017a; Neilson, Avgar, Burton, Broadley, & Boutin, 2018; Rowcliffe, Carbone, Jansen, Kays, & Kranstauber, 2011; Srbek‐Araujo, Chiarello, Srbek‐Araujo, & Chiarello, 2013). These factors should be considered in any CT study when variation in these factors is expected, as they might influence parameter estimates (occupancy, abundance, activity) based on CT data. However, a concise overview of all the factors influencing CT detection of animals and a framework to decide which factors are most important to correct for are lacking. Such a framework should incorporate an explicit consideration of the processes underlying detection of animals by CTs, including ecological processes such as animal abundance and movement, and detection processes, as was called for by Burton et al. (2015). Such a framework will only be effective if it offers practical solutions to correct for biases in detectability that can be used by wildlife managers and conservationists with limited access to both resources and statisticians. Our goal is to provide this framework and rules of thumb when designing or analyzing CT studies.

Here, we present an overview of how (a) animal characteristics (both of individuals and of populations), (b) CT model specifications, (c) CT set‐up protocol, and (d) environmental variables influence detection of animals by CTs within a framework that makes the processes explicit at different scales. This overview can be used to aid study design, correct metrics derived from CT studies, and help select analysis covariates to minimize bias in detection. By pointing toward generic solutions, we hope our framework can be used when there is limited knowledge about the species of interest, or when the CT study is aimed at a whole suite of species.

Lastly, we provide a list of parameters that we think should be measured in each CT study and call for better reporting of these parameters to improve the reuse potential of CT data.

## 2 A CONCEPTUAL FRAMEWORK FOR CT DETECTION

Detection of animals by a CT is a combination of three conditional probabilities. Firstly, we have the probability that an animal moves in front of the CT,

(P1) $$\text{Pr}\left(\text{animal moves past CT}\right).$$

Secondly, comes the probability that an animal triggers the PIR sensor of the CT given that it moved in front of the CT,

(P2) Pr(animal triggers CT|animal moves past CT).

Thirdly, is the probability that an animal is identifiably detected on an image (photograph or video) given that the CT was triggered by its movement in front of the CT,

(P3) Pr(animal identifiably detected|animal moves past CT&triggers CT).

P1 reflects the process of habitat selection of an animal and can be subdivided into the four, spatially nested, orders of selection as originally described by Johnson (1980):

1st order: physical or geographical range of a species [distribution range scale]
2nd order: location of the home range of an individual or social group within the geographic range [landscape scale]
3rd order: usage of habitat components within the home range [habitat patch scale]
4th order: usage of microhabitats such as food items/feeding patches/nest sites/movement trails, etc. within the habitat [microsite scale]

**Figure 1:**
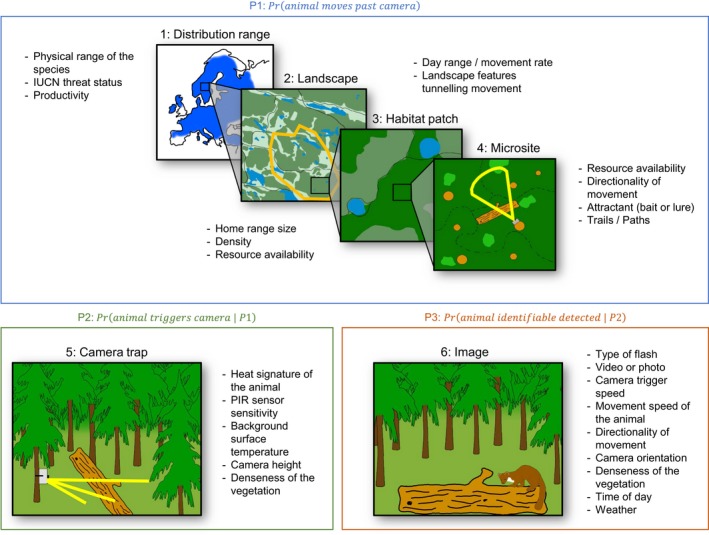
The processes that determine the probability of identifiably detecting an animal species divided into six orders of detection. Four orders at different spatial scales for the probability that an animal passes a CT: 1st order or distribution range scale, 2nd order or landscape scale, 3rd order or habitat patch scale, and 4th order or microsite scale. The 5th order or CT scale for the probability that the animal triggers the PIR sensor of the camera and the 6th order or image scale for the probability that the animal is identifiably detected

We transfer these orders of selection to orders of detection by CTs as follows (see also Figure 1). A CT can only detect an individual of a specific species if it is within the species’ distribution range (1st order or distribution range scale). Within this distribution range, a CT can only detect an individual if it is within the home range of that individual (2nd order or landscape scale). Within that home range, a CT can only detect the individual if it is in a habitat patch that is selected by the animal (3rd order or habitat patch scale). Lastly, within that habitat patch, a CT has a higher likelihood of detecting that individual if it is aimed at a microsite that is selected by the individual (4th order or microsite scale).

To further specify P1, one needs to make explicit what the time frame is for this probability. If we assume a time frame of one day (which is often done in CT studies, e.g., Burton et al., 2015), it becomes the probability that an animal moves past the CT on a given day. This is not only dependent on the above mentioned spatially dependent drivers of P1 but also on temporally dependent drivers such as the distance that an animal travels per day (or day range).

Probabilities P2 and P3 are less influenced by the habitat use of individuals and could be considered within the same hierarchical framework as P1, as subsequently a 5th order for the CT scale (P2), and a 6th order for the image scale (P3), resulting in six orders of detection (Figure 1). Identification of animals at the 6th order can include both the identification of species and the identification of individuals. We have grouped these as we think the same processes influence biases in both types of identification.

## 3 FACTORS BIASING DETECTION OF ANIMALS BY CTS AT DIFFERENT SCALES

Initially, we used our cumulative personal experience of camera trapping in settings as diverse as Norway, Sweden, the Netherlands, South Africa, Myanmar, India, and Turkmenistan to come up with a list of factors that we think directly influence detection of animals by CTs. Based on this list, we performed searches in Scopus during January‐April 2018, using the following search term:

TITLE‐ABS‐KEY ((camera AND trap* OR remote AND camera*)AND (wildlife OR mammal* OR bird*)AND (detection* OR detectability* OR occupancy*)AND (factor of interest)) AND PUBYEAR > 2007

We separately ran a search for each factor by adding it to the search term to reduce the number of papers that needed to be screened. We screened all papers to check if they tested for an effect of the factor on the detection probability, occupancy, or other parameter describing the probability that an animal was photographed by the CT. Furthermore, we used the initially screened papers to expand our list of parameters and ran subsequent searches for those parameters as well. This resulted in a total of 40 variables that we grouped into four groups: 14 animal characteristics (Table 1), nine CT model specifications (Table 2), seven CT set‐up characteristics (Table 3), and 10 environmental variables (Table 4). Note that, we only selected variables that directly influence detection of animals by CTs. Variables that indirectly influence detection of animals by CTs, through one of the listed variables, are not mentioned in the tables but regularly mentioned in the main text when we discuss the selection of covariates.

**Table 1:** Animal characteristics that influence detection by CTs at different orders of detection

Characteristic^a^	Direction and magnitude of effect on detection probability per order^b^						Mechanism	Studies needed	When to correct for^c^	References
	1	2	3	4	5	6				
Day range		++	++	++			Contact with CTs		Species, season, site	(Neilson et al., 2018; Rowcliffe et al., 2008)
Density		++	++	++			Contact with CTs		Species, season, site	(Neilson et al., 2018; Rowcliffe et al., 2008)
Directionality of movement^d^			(−)	(−)	(−)	(−)	Contact with CTs, retention time in front of CT, and identification of detected animals	Combination with other data or (re‐)analysis of CT data	Species, season, site	None
Group size^e^		(+)	(+)	(+)	(+)		Retention time in front of CT	(Re‐)analysis of CT data	Species, season, site	None
Heat signature/surface temperature^f^					++		PIR sensor functionality	Combination with other data or (re‐)analysis of CT data	Species, season, site	(Welbourne et al., 2016)
Home‐range size		++	++	++			Contact with CTs		Species, season, site	(Neilson et al., 2018; Popescu, Valpine, & Sweitzer, 2014; Sollmann et al., 2013; Steenweg et al., 2018)
IUCN threat status/population status^g^	− −						Contact with CTs		Species, site	(Brodie et al., 2015; Cove et al., 2013)
Niche breadth^h^		+	+				Contact with CTs	(Re‐)analysis of CT data	Species, season, site	(Núñez‐Regueiro et al., 2015)
Personality/behavioral responses to CTs				+/−	+/−		Retention time in front of CT		Species, season, site	(Larrucea et al., 2007; Meek, Ballard, Fleming, & Falzon, 2016)
Physical or geographical range of the species	++						Contact with CTs	Combination with other data or (re‐)analysis of CT data	Species, season, site	(McDonald et al., 2015)
Speed of movement					−	−	Retention time in front of CT and identification of detected animals	Combination with other data	Species, season, site	(Rowcliffe et al., 2011)
Taxonomy^i^						−	Identification of detected animals	Combination with other data or (re‐)analysis of CT data	Species	(Welbourne, MacGregor, Paull, & Lindenmayer, 2015)
Territoriality^j^							Contact with CTs	Combination with other data or (re‐)analysis of CT data	Species, season, site	(Steenweg et al., 2018)
Time spend on the ground^k^			+	+	+		Contact with CTs and retention time in front of CT	Combination with other data or (re‐)analysis of CT data	Species, season, site	(Rovero, Martin, Rosa, Ahumada, & Spitale, 2014)

a Characteristics are seen as continuous variables unless otherwise stated in the table or the footnotes, where the direction of the effect given is with an increase in the characteristic. For example, detection probability increases with an increase in day range.

b Direction and magnitude of effect on detection probability given in a scale from ++ to − − with 0 if no effect was found, biases given between brackets are not based on literature but estimates from the authors. When multiple studies reported contrasting results, we give the reported range separated with a /.

c Factor given needs to be corrected for if multiple of these are considered in a study (see main text).

d Directionality of movement can be considered at different spatial scales and is compared to a more tortuous movement. At the 3rd and 4th order an increase in directionality of the movement lowers the probability of an animal encountering a camera trap (at fixed day range). Similarly, at the 5th order an increase in directionality lowers the retention time in front of the CT (less distance is covered in front of the CT), lowering the probability of capture. At the 6th order an increase in directionality lowers the potential for multiple pictures at different angles of the same individual, reducing the probability that the individual can be identified to species or individual. Directionality of movement can differ between species and seasons, but also between sites due to differences in food availability or landscape configuration (see Table 4).

e If animals move in groups, the probability that one individual triggers the CT and any individual from the group remains in the field of view of the CT increases. This is similar to an increased detection probability with group size in distance sampling (Buckland et al., 2001).

f Detection probability increases with increasing difference in surface temperature of the animal versus surface temperature of the surroundings and detection probability increases with increasing surface area of the animal.

g IUCN threat status is determined by a combination of the change in geographical range of a species and a change in population size of a species (IUCN, 2018). The threat status increases as geographical range and/or population size decline. Therefore, regardless of the current geographical range and population size, detection probability at the 1st order decreases with increasing threat status.

h Species with a larger niche breadth have a higher probability of walking past randomly placed CTs. However, when targeting CTs for a specific species, the detection probability will be higher when the species has a smaller niche breadth, as these species can be more effectively targeted.

i Detection decreases (misidentification increases) with increasing number of related species co‐occurring in the same area.

j When animals use their territory exclusively, this reduces the number of individuals present in a home range and thus detection probability at the 2nd order. Territoriality can differ between species, seasons, and sites depending on species traits and resource availability.

k Time spent on the ground in relation to CTs placed at ground level. This relationship is reversed when CTs are deployed somewhere else. This could be to target semi‐aquatic or semi‐arboreal species by placing CTs, respectively, above water or in the forest canopy (e.g., Bowler, Tobler, Endress, Gilmore, & Anderson, 2017; Swinnen, Hughes, & Leirs, 2015).

**Table 2:** CT model specifications that influence detection by CTs at different orders of detection

Characteristic^a^	Direction and magnitude of effect on detection probability per order^b^						Mechanism	Studies needed	When to correct for^c^	References
	1	2	3	4	5	6				
Battery level^d^					+	+	PIR sensor functionality, identification of detected animals		Design, season	(Meek & Pittet, 2012; Rovero et al., 2013)
Battery type^e^					+/−	+/−	PIR sensor functionality, identification of detected animals		Design, season	(Meek & Pittet, 2012; Rovero et al., 2013)
Camera lens focal length^f^					(−)	(+/−)	Retention time in front of CT and identification of detected animals	Application of multiple CT models	Design	(Meek & Pittet, 2012)
Image resolution						+/−	Identification of detected animals		Design	(Meek & Pittet, 2012; Rovero et al., 2013)
Infrared or white flash^g^					0/−	+/−	Identification of detected animals		Design, season	(Glen, Cockburn, Nichols, Ekanayake, & Warburton, 2013; Rovero et al., 2013)
PIR sensor angle^h^					+	(−)	PIR sensor functionality		Design	(Meek & Pittet, 2012; Rovero et al., 2013)
PIR sensor sensitivity					++		PIR sensor functionality		Design	(Meek & Pittet, 2012; Rovero et al., 2013)
Trigger speed of the CT						+	Identification of detected animals		Design	(Fancourt, Sweaney, & Fletcher, 2018; Meek & Pittet, 2012; Rovero et al., 2013)
Type of resources (video or photographs)^i^						+	Identification of detected animals		Design	(Meek & Pittet, 2012; Rovero et al., 2013)

a Characteristics are seen as continuous variables unless otherwise stated in the table or the footnotes, where the direction of the effect given is with an increase in the characteristic. For example, detection probability increases with an increase in trigger speed.

b Direction and magnitude of effect on detection probability given in a scale from ++ to − − with 0 if no effect was found, biases given between brackets are not based on literature but estimates from the authors. When multiple studies reported contrasting results, we give the reported range separated with a /.

c Factor given needs to be corrected for if multiple of these are considered in a study (see main text). Design refers to studies using a study design in which multiple CT models are used.

d PIR sensor sensitivity and flash intensity decrease with battery level.

e Different types of batteries (lithium, NiMH, NiZn, and alkaline) have different voltage specifications and have different discharge curves influencing PIR sensor sensitivity and potentially flash intensity over time.

f The focal length of the camera lens determines the size of the field of view (a lower focal length results in a larger field of view). Therefore, we argue that a longer focal length reduces the retention time of an animal in front of the CT as the field of view is smaller. Furthermore, it could result in increased identification of species or individuals further away (as these will be larger in the frame) while at the same time it would decrease identification of animals closer to the CT as they might end up partly outside of the frame.

g Many animals respond negatively to white flash (either xenon or LED) thus reducing retention time in front of the CT and the likelihood of the animal being recorded. However, if an animal is recorded, the quality of the image is often much better with white flash (best with xenon flash). Due to responses to the flash, the likelihood of obtaining multiple images is however lower, which might reduce the potential for good species or individual identification. The effect of the flash can differ between seasons due to differences in day length and the fact that the flash is only used at night.

h The number of triggers of animals outside of the field of view of the camera increases with PIR sensor angle, decreasing detectability at the 6th order.

i Most CTs can either take single photographs, a burst of photographs, or video. The more material is collected, going from single photographs to a burst of photographs to video, the higher the probability of species or individual identification (6th order) as behavior and multiple angles can aid identification. There is, however, a trade‐off as most CTs have a lower trigger speed when using video compared to photo mode.

**Table 3:** Study set‐up characteristics that influence detection by CTs at different orders of detection

Characteristic^a^	Direction and magnitude of effect on detection probability per order^b^						Mechanism	Studies needed	When to correct for^c^	References
	1	2	3	4	5	6				
CT density		+	+	+			Contact with CTs		Design	(Foster & Harmsen, 2011; Smith, Legge, James, & Tuft, 2017)
CT height^d^					0/− −	(+/−)	PIR sensor functionality		Design	(Jacobs & Ausband, 2018; Meek, Ballard, & Falzon, 2016)
CT orientation (angle relative to the ground)^e^					(+/−)	(+/−)	PIR sensor functionality and identification of detected animals	Application of multiple CT setups	Design	None
CT orientation (horizontal or vertical)					+/−	+/−	PIR sensor functionality and identification of detected animals	Application of multiple CT setups	Design	(Smith and Coulson, 2012)
CT orientation (relative to the sun)^f^						(+/−)	Identification of detected animals	Application of multiple CT setups	Design, Season	None
Duration of deployment		++	++	++			Contact with CTs		Design	(Larrucea et al., 2007; Smith et al., 2017; Stokeld et al., 2016)
Number of CTs per trapping station		+	+	+	+	+	Contact with CTs, retention time in front of CT, and identification of detected animals		Design	(O'Connor et al., 2017; Smith et al., 2017; Stokeld et al., 2016)

a Characteristics are seen as continuous variables unless otherwise stated in the table or the footnotes, where the direction of the effect given is with an increase in the characteristic. For example, detection probability increases with an increase in CT density.

b Direction and magnitude of effect on detection probability given in a scale from ++ to − − with 0 if no effect was found, biases given between brackets are not based on literature but estimates from the authors. When multiple studies reported contrasting results, we give the reported range separated with a /.

c Factor given needs to be corrected for if multiple of these are considered in a study (see main text). Design refers to studies using multiple study designs.

d The distance between the animal and the CT increases with increasing CT height, potentially resulting in better (for close animals) or worse (for animals further away) identification of species and individuals.

e Changing the angle of the CT might change PIR sensor functionality (due to the targeted Fresnel lens: Welbourne et al., 2016), and at the 6th order, it might influence the ability to identify species or individuals due to a changed perspective.

f Although several studies mention that direct sunlight can reduce visibility and thus identification of species or individuals (e.g., Meek et al., 2014), we could not find any study testing for an effect of CT orientation relative to the sun on detection probability.

**Table 4:** Environmental variables of CT location that influence detection by CTs at different orders of detection

Characteristic^a^	Direction and magnitude of effect on detection probability per order^b^						Mechanism	Studies needed	When to correct for^c^	References
	1	2	3	4	5	6				
Attractant (bait or lure)^d^			+	++/−	++/−		Contact with CTs and retention time in front of CT		Design, season	(Diete, Meek, Dixon, Dickman and Leung, 2016; Satterfield et al., 2017; Suárez‐Tangil and Rodríguez, 2017)
Background temperature^e^		−	−	−	− −		Contact with CTs and PIR sensor functionality		Season, site	(Nagy‐Reis et al., 2017; Lesmeister et al., 2015; Pease, Nielsen and Holzmueller, 2016; Welbourne et al., 2016)
Denseness of the vegetation		−	−	− −	− −	− −	Contact with CTs, PIR sensor functionality, and identification of detected animals		Design, season, site	(Hofmeester et al., 2017a; Rich et al., 2016)
Distance of animal to the camera					− −	− −	PIR sensor functionality and identification of detected animals		Design, season, site	(Hofmeester et al., 2017a; Howe et al. 2017; Rowcliffe et al., 2011)
Human disturbance^f^		+/−	+/−	+/−	(−)	(−)	Contact with CTs		Season, site	(Larrucea et al., 2007; Wearn et al., 2017)
Landscape features channeling animal movement (e.g., trails)			++	++			Contact with CTs		Design, season, site	(Cusack, Dickman, et al., 2015a; Harmsen et al., 2010; Kolowski and Forrester, 2017; Reilly, Tobler, Sonderegger and Beier, 2017; Srbek‐Araujo et al., 2013)
Repulsive features in the landscape^g^			−	−	−		Contact with CTs and retention time in front of CT		Design, season, site	(Khorozyan et al., 2014; Larrucea et al., 2007; Mann, O'Riain, & Parker, 2015; Nagy‐Reis et al., 2017; Rich et al., 2017)
Resource availability	++	++	++	++	++		Contact with CTs and retention time in front of CT		Design, season, site	(Brassine & Parker, 2015; Lesmeister et al., 2015; Nagy‐Reis et al., 2017; Rich et al., 2017)
Time of day (day vs. night)^h^						+/−	Identification of detected animals		Season, site	(Cusack, Swanson et al., 2015b; Nagy‐Reis et al., 2017; Rowcliffe et al., 2014)
Weather					+/−	+/−	PIR sensor functionality and identification of detected animals		Season, site	(Lesmeister et al., 2015; Nagy‐Reis et al., 2017; Pease et al., 2016)

a Characteristics are seen as continuous variables unless otherwise stated in the table or the footnotes, where the direction of the effect given is with an increase in the characteristic. For example, detection probability increases with an increase in resource availability.

b Direction and magnitude of effect on detection probability given in a scale from ++ to − − with 0 if no effect was found, biases given between brackets are not based on literature but estimates from the authors. When multiple studies reported contrasting results, we give the reported range separated with a /.

c Factor given needs to be corrected for if multiple of these are considered in a study (see main text). Design refers to studies using multiple study designs.

d Attractants can have different effects on different species depending on the type of attractant and the species life history, for example, using meat as attractant will most likely attract carnivores but not necessarily ungulates or other herbivores.

e Presented direction of bias is for endotherms, as for cold ectotherms, the relationship is reversed (they are better detected at higher background temperatures). The effect of temperature is both due to avoidance by animals of the hottest parts of the landscape (2nd‐4th order) and due to the influence of background temperature on the PIR sensor functionality (5th order)

f Human disturbance can have a positive or negative bias depending on how well individuals in a population/of a given species are adapted to human disturbance. Furthermore, humans can damage or sabotage CTs which leads to a negative bias at the 5th and 6th order.

g Repulsive features in the landscape are often human features, such as a highway, that reduce detection of certain species. There are however large differences between species in terms of being repulsed or attracted to the same landscape features (see cited references).

h For most species, species and individuals can be better identified using color images (at day or with white flash) than using black and white images (infrared flash). Furthermore, the range of the flash decreases identification probability at night, while this is not the case with natural (day) light.

To estimate the direction and magnitude of the effect for all factors, we used the parameter estimates of the studies that included the factor to qualitatively determine the direction and magnitude of the effect on detection probability. We only included factors that directly influenced one of the following mechanisms (Tables 1, 2, 3, 4): contact with CTs (1st–4th order), retention time in front of CT and PIR sensor functionality (5th order), and identifiable detection of animals or individuals on images (6th order). The key papers reporting direction and magnitude for factors are referred to in Tables 1, 2, 3, 4. Note that, the reported biases do not necessarily show linear relationships with detection probability.

For factors in our initial list for which we did not find any references in the literature, we used our experience and knowledge about the different processes affecting detection to estimate the likely direction and effect size, for which we provide our reasoning in the footnotes of Tables 1, 2, 3, 4. For these factors, and factors where we only found one study testing the effect, we also provide a suggestion for what kind of studies need to be done in order to identify the direction and magnitude of the effect on detection (Tables 1, 2, 3, 4). Thus, we did not do a formal systematic review but rather used the search results and information from the resulting papers complemented by our own experience and ideas to derive as complete a list as possible.

## 4 AVOIDING OR CORRECTING FOR BIASES IN DETECTION

Biases (especially those in Tables 2 and 3) can be partially avoided by standardizing the CT set‐up protocol and CT model that are used in a study. When this is not possible, for example, when using data from multiple studies or when studying multiple species, seasons, or sites, there are two ways of correcting for these biases: by correcting the metric of interest or by using covariates in a statistical framework.

### 4.1 Correcting the metric of interest

Several different metrics can be derived from CT data of which one, photographic capture rate (number of passages per unit of time), can be corrected for biases directly. This capture rate is often used as a relative abundance index (RAI: Carbone et al., 2001) or as a measure of patch use (Hofmeester, Rowcliffe, & Jansen, 2017b). There has been a lot of critique of this metric as it is potentially heavily biased by differences in detectability at different scales (Anile & Devillard, 2016; Sollmann, Mohamed, Samejima, & Wilting, 2013). Some of these biases, in particular those at the 5th order, can, however, be dealt with by quantifying the effective detection range of CTs (Hofmeester et al., 2017a; Rowcliffe et al., 2011). In short, distance sampling methodology can be used to estimate the effective detection distance and angle of the CT for each species, habitat type or season, or they can be estimated using covariates (see below for which covariates to select). These estimates can then be used to correct the capture rate for differences in detectability at the 5th order, yielding a corrected index that reflects microsite use and is comparable among species, sites, and seasons. This index can thus only be used as an estimate of microsite use (e.g., Hofmeester et al., 2017b) and not as a (relative) density index unless differences in movement rates are also corrected for (sensu Rowcliffe, Field, Turvey, & Carbone, 2008).

### 4.2 Using covariates in a statistical framework

Instead of correcting the metric directly for biases, it is also possible to correct for biases by considering specific covariates in a statistical framework. These can range from relatively simple multiple linear regression of photographic capture rates to hierarchical models with multiple hierarchical levels (Anile & Devillard, 2016; Mordecai, Mattsson, Tzilkowski, & Cooper, 2011). The disadvantage of simple multiple linear regression is that single processes that influence both detection and the ecological process that is the aim of the study cannot be separated.

The detection and ecological processes sampled by CTs can be separated statistically using hierarchical models that make processes at different scales explicit (Kéry & Royle, 2016). It is thus possible to model each of the six scales we identified separately in a hierarchical model. The occupancy models often used with CT data are a good example of a hierarchical model in which the ecological or state process (occupancy or patch use) and the detection process are modeled separately (MacKenzie et al., 2006). Other hierarchical models include the Royle–Nichols model (Royle & Nichols, 2003), spatially explicit capture–recapture (SCR) models (Efford, 2004; Royle, Karanth, Gopalaswamy, & Kumar, 2009) and distance sampling (Buckland et al., 2001; Howe, Buckland, Després‐Einspenner, & Kühl, 2017). As the effect of covariates on the hierarchical processes are estimated using different parts of the data—for example, the number of visits at which a species was detected for the detection probability, and the number of sites with and without detections for the occupancy part of an occupancy model—these models can separate effects of the same covariate on the different processes. All of these models have been applied to CT data, and all allow the use of covariates for the different parts of the model.

When applying hierarchical models to CT data, it is important to consider the spatial, as well as the temporal scale at which inference is made (Efford & Dawson, 2012; Steenweg, Hebblewhite, Whittington, Lukacs, & McKelvey, 2018). After selecting an appropriate statistical framework, the next step is to select the proper covariates at different hierarchical levels in the model.

## 5 HOW TO SELECT THE APPROPRIATE COVARIATES?

It would be impractical and statistically impossible to always correct for all 40 factors presented in Tables 1, 2, 3, 4. It might also not be necessary, as it depends on the specific aims and design of the study whether variation in these factors is to be expected (Table 5). Considering two examples, a study investigating the visitation frequency of herbivores in relation to damage to forestry (interest in 4th order habitat selection) would only need to correct for unwanted bias due to factors at the 5th and 6th order. This is because the nature of the question makes it irrelevant whether it is selection of individuals at the 2nd, 3rd, or 4th order that determines these visitation rates and the correction is only needed to get an unbiased estimate of visitation by the species.

**Table 5:** Relationship between the aim of the study and the potential scales at which biases need to be considered

Aim of the study	Which scales need to be considered
Species distribution	2nd–6th
Species richness/biodiversity	2nd–6th
Abundance/density	3rd–6th
Community ecology/species interactions	3rd–6th
Population demography	3rd–6th
Activity pattern	4th–6th
Behavioral	5th and 6th
Patch use/local activity	5th and 6th

In contrast, a study investigating the species richness of a set of nature reserves (interest in 1st/2nd order habitat selection) would need to correct for unwanted species and site‐specific biases due to factors at the 2nd–6th order. This is because different species might be more or less easy to identify (6th order), have different heat signatures influencing PIR sensor sensitivity (5th order), have differences in microsite (4th order) and habitat selection (3rd order), or have different densities (2nd order). These issues might not only differ between species, but also between sites for a single species, and there might be interactions between factors, for example, differences in densities of two related species between sites (2nd) might increase the bias due to misidentification (6th order).

**Figure 2:**
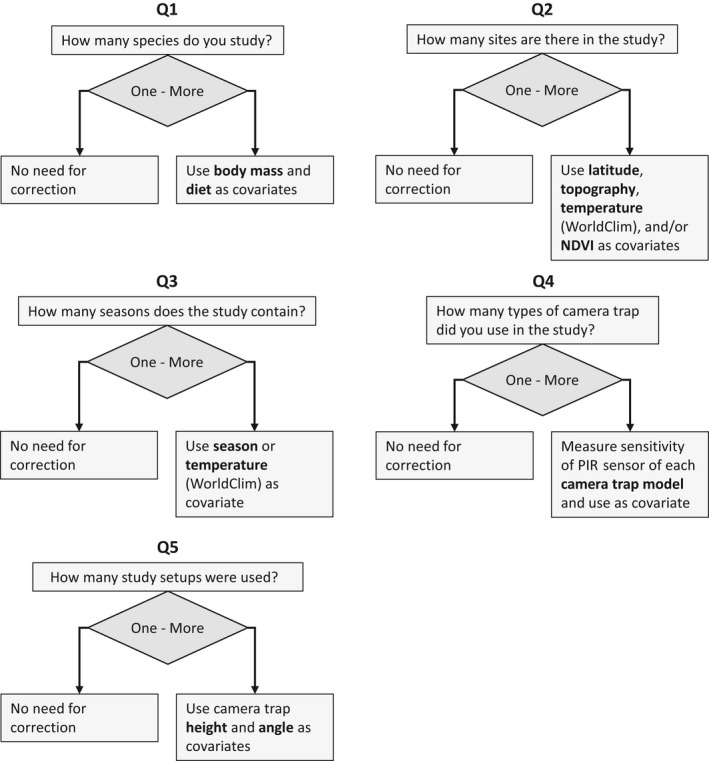
Questions that lead to selection of covariates for correction in detection. When performing a CT study or when analyzing CT data, the following questions should be asked in relation to differences in detectability. For each question where the answer is multiple, an effort needs to be made to analyze or correct for potential biases related to this parameter as presented in the main text

Similarly, a study investigating the abundance or habitat use of one species in one site would not need to correct for differences among species or sites. We came up with a set of simple questions (Figure 2) to determine which group of factors needs to be considered under different study scenarios. These groups are also presented in Tables 1, 2, 3, 4. For each group, we present one or several overarching parameters that are correlated to the factors in Tables 1, 2, 3, 4, making correcting for the detection process simple and effective. Combining Table 5 and Figure 2 shows that factors affecting detection at the 5th and 6th order need to be considered in most studies, making the 5th order the most important one to consider as most biases at the 6th order (Tables 2 and 3) can relatively easily be accounted for by standardizing CT model and CT set‐up protocol. For example, using a single CT model with the same settings and a standardized set‐up protocol will correct for biases due to type of flash, type of image (video or photograph), camera trigger speed, and camera orientation. As CT studies often investigate research questions at the 2nd or 3rd order (Table 5), there is often a need to also correct for 4th order biases. Combining the above, it seems most important to correct for 4th and 5th order (microsite and camera trap) biases in most CT studies.

### 5.1 Multiple species

When considering multiple species within the same site in a CT study, one needs to correct for differences among the species for the factors given in Table 1 and for differences in responses of species to factors in Table 4. Many of the factors in Table 1 are related to a few basic life‐history traits of animals. At the 2nd–4th orders, home‐range size and day‐range/movement rates of mammals are scaled with body mass and diet (Carbone, Cowlishaw, Isaac, & Rowcliffe, 2005; Tucker, Ord, & Rogers, 2014), as are directionality and speed of movement (Rowcliffe, Jansen, Kays, Kranstauber, & Carbone, 2016). Also, the response of species to resource availability in the landscape (Table 4) is determined by diet and body mass of mammals (Fisher, Anholt, & Volpe, 2011), and body mass scales with density with variations in the scaling due to diet (Carbone, Rowcliffe, Cowlishaw, & Isaac, 2007). Digestive physiology further influences the spatial distribution of herbivores as nonruminants are more evenly distributed over the landscape compared to ruminants of similar size (Cromsigt, Prins, & Olff, 2009).

At the 5th order, the detection of animals when walking in front of the CT is determined by the functionality of the PIR sensor of the CT, which is mainly determined by the heat signature of the animal, that is, the difference in temperature between the surface of the animal and the background surface temperature (Welbourne, Claridge, Paull, & Lambert, 2016). This heat signature and the related detection zone are again a function of body mass (Hofmeester et al., 2017a; Rowcliffe et al., 2011). Concluding, most factors in Table 1 scale with body mass dependent on diet type. Therefore, these two life‐history traits are important candidates as covariates in models of the detection process (e.g., Cove, Spínola, Jackson, Sàenz, & Chassot, 2013). Similarly, when using the photographic capture rate, body mass and diet could be included as covariates in a multiple regression model to correct for detection differences (Anile & Devillard, 2016) or when modeling the effective detection distance and angle (Hofmeester et al., 2017a; Rowcliffe et al., 2011). The fact that body mass is scaled with so many parameters in Table 1 might make it difficult to disentangle the different processes that body mass influences. Depending on the question, this could be a disadvantage, but it could also be an advantage as one could correct for multiple biases with one parameter.

Some other parameters might need to be considered depending on the species. For example, differences among species in group size and the time spent on the ground (as opposed to being arboreal, aquatic, subnivean, or fossorial) could potentially have a large influence on detection probability. Especially, if part of the species community is (semi)arboreal, (semi)fossorial, or (semi)aquatic, this will highly influence detection probability although the number of studies investigating this bias is very limited.

Most CT studies are designed to target a specific species (often a large carnivore) after which the “by‐catch” data are used to study whole communities (Harmsen, Foster, Silver, Ostro, & Doncaster, 2010; Rich et al., 2016; Tobler et al., 2008). In such studies, the setup of the CTs for the primary target influences detection probability of other species. Most likely, the targeted species will be overrepresented (Anile & Devillard, 2016), while other species such as prey of the targeted species will be underrepresented (Harmsen et al., 2010). These kinds of differences in detection probability among species can, to some extent, be corrected for by estimating a species‐specific detection probability using hierarchical models (Efford & Dawson, 2012; Royle & Nichols, 2003).

Many of the variables in Table 1 are also different for the different sexes (or demographic groups such as females with dependent young) within one species. Therefore, if the aim of the CT study is to derive estimates of sex ratio or the demography of a population, detectability differences between the sexes need to be taken into account (Singh, Qureshi, Sankar, Krausman, & Goyal, 2014; Srbek‐Araujo, 2018). Similarly, if the aim of the study is to derive densities based on the recognition of individuals, such as (spatial) capture–recapture, differences in detectability between sexes, or demographic groups need to be taken into account (Larrucea, Brussard, Jaeger, & Barrett, 2007). Also, in these studies, differences between camera traps at the 6th scale become more important as image quality becomes an important determinant of the possibility of identifying individuals.

### 5.2 Multiple sites/seasons

When considering multiple sites or multiple seasons in a CT study, one needs to consider a variety of factors related to both animal characteristics and environmental variables that might differ among the sites/seasons. Many of the factors in Table 1 are dependent on season or site variables. For example, at the 2nd order, the home‐range size of individuals of a species is expected to be different among sites and seasons due to differences in resource needs and energy expenditure (McNab, 1963). Movement parameters can vary within species, often along gradients of environmental productivity (Duncan, Nilsen, Linnell, & Pettorelli, 2015) such that remote sensing products like NDVI can be used to correct for some of the expected inter‐site variation. Similarly, at the 5th order, the heat signature of the animal will be different in different seasons and sites due to better insulation of a winter coat compared to a summer coat (Hart, 1956) and differences in background surface temperature among seasons, sites, and even CT locations. Season also influences weather, temperature, and the denseness of the vegetation (Lesmeister, Nielsen, Schauber, & Hellgren, 2015; Nagy‐Reis et al., 2017; Rowcliffe et al., 2011). Weather can have direct effects on detection by CTs: snow, ice, or rain can block the field of view of the camera; snow, ice or rain on the sensor can lower the sensitivity of the PIR sensor; and low temperatures drain the batteries quicker (Cho, Choi, Go, Bae, & Shin, 2012). The majority of seasonal differences could be corrected for by simply adding season as a covariate in whichever model is used to analyze the data. Otherwise, site‐specific estimates of temperature and precipitation using global datasets using local weather stations such as WorldClim (Fick & Hijmans, 2017) can be used as covariates.

A simple solution using a factor such as season is less convenient when dealing with different sites as the number of levels in the factor could easily become very large when many sites are surveyed. In that case, overarching parameters such as altitude, latitude, climatic region, NDVI, or percentage forest cover might be useful to classify sites. Several studies have classified land use in distinct classes and used those as a covariate in models (Ehlers Smith, Ehlers Smith, Ramesh, & Downs, 2018; Nagy‐Reis et al., 2017; Rich et al., 2016; Wearn et al., 2017). We, however, advise the use of a continuous variable such as NDVI (as an index of environmental productivity), or extent of forest cover obtained from satellite images as overarching parameters that influence most parameters in Table 4. Latitude influences day length, weather conditions, and other variables that are related to detection but has to our knowledge not been used as a covariate to deal with these issues yet. Altitude and climatic region influence factors like weather, background surface temperature, and resource availability and could be used as covariates, although the latter will mainly be important for global scale studies. When large differences in background surface temperature are expected among study sites, satellite‐derived global estimates of surface temperature per site might be used to correct for differences in 5th order detection probability. Similarly, the temperature as directly recorded by many CT models could be used as a covariate dealing with this issue. In this case, care has to be taken as to the reliability of these measurements as direct sunlight on the CT might result in too high measurements (Meek et al., 2014). So far, we are not aware of any studies using such an approach.

Lastly, correcting for biases becomes more problematic when dealing with multiple seasons/sites and multiple species as factors might show interactions. For example, changes in home‐range size between seasons might be different for species with different body mass and/or diets (McNab, 1963). In such circumstances, it becomes even more important to reduce the number of parameters to a minimum, to make the exploration of interaction terms between parameters in statistical models possible.

### 5.3 Multiple CT models

When considering multiple CT models in the same study, one should consider the factors described in Table 2. This problem will especially arrive in long‐term studies as newer CT models replace the old ones (Rovero et al., 2013). Similarly, newer versions of the same model could potentially also have undergone changes in the angle and sensitivity of the PIR sensor or other characteristics. A simple solution would be to use CT model as a covariate if more than one model is used (Kelly & Holub, 2015). Alternatively, as the PIR sensor angle and sensitivity are likely the most influential CT model parameter determining detection, these parameters can be measured for the different CT models in a regulated environment. One could, for example, measure the distance and angle at which each CT model is triggered by a warm object moving at a fixed speed (Swann, Hass, Dalton, & Wolf, 2004). These measurements can then be used as continuous covariates in the statistical model.

### 5.4 Multiple setups

Most single studies will not use different setups unless the aim of the study is to compare the different setups. Therefore, the problem of multiple setups will mainly arise when combining data that were collected during different studies (Scotson, Johnston, et al., 2017b). Unfortunately, there is no simple solution when it comes to correcting for differences in setup. The most important issue to consider is the placement of CTs, specifically if they are aimed at a specific object (such as a trail or behaviorally important feature such as a scent‐marking location) or placed at a predefined location regardless of small‐scale landscape features. Most single‐species studies use a directed placement to increase the detection probability for the target species (Harmsen et al., 2010). Camera trap placement is especially important when considering multiple species, as described above, but also becomes important when studying a single species using data from multiple studies as the detectability of the same species is different for random versus nonrandom placed CTs (Cusack, Dickman, et al., 2015a). Therefore, inference made from multiple studies using different setups should be taken with care and where possible should at least use the type of setup as covariate in a statistical model.

Another important issues related to CT setup is the use of an attractant (either bait or a lure) or not. The advantages and disadvantages of using attractants are thoroughly discussed previously (Zimmermann & Foresti, 2016). In short, the use of an attractant might change the spatial behavior and distribution of animals violating assumptions of several statistical models such as the random encounter model (Rowcliffe et al., 2008), occupancy models, or spatially explicit capture–recapture (Gerber, Karpanty, & Kelly, 2012). Specifically, the use of bait can increase recapture probability, which should be incorporated in the statistical framework (Gerber et al., 2012; du Preez, Loveridge, & Macdonald, 2014). Baiting can also have negative impacts when species become accustomed to this form of supplementary feeding (Balme et al., 2014). We thus recommend to carefully consider the use of bait and to correct for potential biases due to baiting when analyzing CT data.

A third issue related to CT setup that is important to consider is the spacing of CTs or CT density. This is especially important when combining data from different studies to estimate occupancy or densities in different areas as several models estimating these variables require different setups. For example, models estimating density based on SCR require a dense grid of CTs with several CTs per home range enabling the capture of single individuals on multiple CTs (O'Brien, 2011). Contrastingly, models estimating occupancy assume independence between sites, necessitating a less dense spacing of camera traps with a maximum of one per home range if each CT is considered a separate site in the occupancy model (O'Connell & Bailey, 2011). Camera spacing should thus be considered when choosing a model for comparisons between studies. Subsampling of CTs could be used to reduce CT density if needed for between‐study comparisons.

Several other variables related to CT setup that might affect detection have not been studied yet (Table 3), so there is a need for a better understanding of how these variables influence detection. A simple way to study these effects is by comparing studies using multiple setups from a limited geographic region in the same season. For this purpose, it is very important that studies report all factors related to the CT set‐up protocol (CT height, CT orientation, etc.) in a standardized way (see below).

## 6 INCORPORATING DETECTION BIAS AT DIFFERENT SCALES INTO DESIGN OF CT STUDIES

Our adaptation of the framework of Johnson (1980) can also be used to minimize detection bias at different scales while designing CT studies. This is implicitly already done by many studies targeting large carnivores (e.g., Harmsen et al., 2010), but we think it would be good to make this incorporation explicit. When targeting a single species, knowledge of the distribution and habitat selection of that species on the 1st–4th orders can be used to select microsites in the landscape where the probability of detection is maximized. For example, when it is known that a certain species uses roads (microsite selection at the 4th order) as a travel route between foraging sites (sites that are selected on the 3rd order), CTs can be placed on roads in these areas to increase detectability. Placing CTs on sites that are selected by the target species at the 4th order reduces problems with detection probability to only 5th and 6th order factors. Note that, when using such a strategy, the interpretation of the data should be based on the presence of animal movement and not necessarily the intensity of local space use or animal density (Stewart, Fisher, Burton, & Volpe, 2018).

In multi‐species studies, knowledge about habitat use of different species can be used to select sites with relatively equal probabilities of all species being present, which is often the aim when performing random CT placement.

## 7 THE IMPORTANCE OF STANDARDIZED REPORTING

The amount of CT data is increasing, and so does the wish for using this data for comparative studies (Scotson, Johnston, et al., 2017b; Steenweg et al., 2017). This calls for a better standardization of data collection and the reporting of data, which is essential for ecological data to be reused (Zimmerman, 2008). There are several recent initiatives to facilitate standardization, such as the guiding principles by Meek et al. (2014), the GBIF best practise guide by Cadman and González‐Talaván (2014), and the CT Metadata Standard (Forrester et al., 2016). Here, we list the most important factors identified by these three documents that we think should be reported to be able to correct for biases in detection.

- What is considered an independent event/sequence
- Length of the survey, of each CT deployment and actual sampling effort (number of days the camera was active)
- CT model and settings (quiet period, sensor sensitivity, trigger speed, photograph, burst of photographs or video, type of flash, etc.)
- Coordinates of deployment (latitude and longitude in decimal degrees using datum WGS84)
- Use of bait/lure, if used, which bait/lure, distance between bait/lure and CT, and how often it was renewed
- Placement of CT (along specific features, such as trails, roads, or waterpoints; systematic or random; number of CTs per station, etc.)
- Temperature and weather during the survey
- Number and spacing of CTs
- Height of the CT, angle to the ground, and CT orientation
- Vegetation density and habitat modification in front of the CT

## 8 CONCLUSION

We present an overview of the factors that influence CT detection of animals. Overall, we believe that factors at the 4th and 5th order result in the largest biases (Tables 1, 2, 3, 4), although we would strongly encourage investigators to explicitly test this under a variety of circumstances. Factors that influence detection at these scales are therefore most important to correct for in CT studies. We think that our framework will clarify the functioning of CTs, by making the processes at different scales explicit. Furthermore, we hope it will aid in (a) the design of CT studies, (b) the correction of CT metrics, and/or (c) the selection of covariates to decrease unwanted bias in CT data. Lastly, we hope our framework will contribute to making CT methodology more robust.

We hope to encourage the study of factors affecting detection of animals by CTs and reporting of field experience and model outcomes that can aid in furthering CT methodology. We want to stress that, when designing CT studies, it is better to avoid introducing bias in your data than to correct for it afterward. This means standardization of the CT set‐up protocol and the CT model used. Which factors to take into account when designing a study depends on the study question. As such, there are no simple guidelines, which make reporting of studies in a repeatable way even more important.

Last, we hope that providing a conceptual framework to deal with issues of detectability by CTs aids in a quality improvement of CT studies enabling the (re)use of data for global studies of mammal communities. Especially, when it comes to the (re)use of data from multiple studies, it might be impossible to account for some biases in the data. In such cases, extra care should be taken when interpreting the results and potential biases discussed.

## CONFLICT OF INTEREST

The authors declare that they have no conflict of interest.

## AUTHORS’ CONTRIBUTIONS

TH, JC, and JL conceived the study, TH wrote the draft manuscript, all authors contributed to the conceptual framework and the list of factors determining CT detection of animals. All authors read and approved the final manuscript.

## Data Availability

All data used in this review are presented in the manuscript.
